# Suggestion of an Improved Evaluation Method of Construction Companies’ Industrial Accident Prevention Activities in South Korea

**DOI:** 10.3390/ijerph18168442

**Published:** 2021-08-10

**Authors:** Sung-Yong Kang, Seongi Min, Deokhee Won, Young-Jong Kang, Seungjun Kim

**Affiliations:** 1 School of Civil, Environmental and Architectural Engineering, Korea University, Seoul 02841, Korea; jokeksy@korea.ac.kr (S.-Y.K.); tjsrl541@korea.ac.kr (S.M.); yjkang@korea.ac.kr (Y.-J.K.); 2 Maritime ICT R&D Center, Korea Institute of Ocean Science and Technology, Busan 49111, Korea; thekeyone@kiost.ac.kr

**Keywords:** construction safety, pre-disaster prevention, occupational safety and health policy, policy and institutional research, questionnaire

## Abstract

Workers in the construction industry are constantly exposed to dangers during work that can lead to death or disability. Despite recent advances in construction technology, the presence of these risks for workers has become an unresolved social problem. In particular, most companies often recognize that it is necessary to mitigate against risks posed to worker only after an accident has occurred. Recently, there has been an increasing demand for the development of new safety technologies and policy proposals to ensure the safety of workers during construction or work. However, the right solution is not coping after an accident but preventing it, and this must be accompanied by voluntary efforts by the company. To work toward such solutions, Korea is implementing an evaluation of construction companies’ industrial accident prevention activities without legal regulations or coercion to encourage voluntary accident prevention activities by companies. The purpose of this study is to propose an effective improvement direction for the system implemented by the Korea Occupational Safety and Health Agency. First, by analyzing the details of the system and the data of the evaluation results, the system’s effectiveness and rationality are reviewed, and steps for improvement are determined. Next, an evaluation model is proposed considering the size of the company to be evaluated and the level of safety and health, and its validity is verified through a survey of construction workers. Finally, a plan to induce the voluntary participation of construction companies in this system and the role of the supervisory authority are presented. This study is expected to serve as an important example of an effective safety policy model by encouraging companies’ voluntary efforts to prevent accidents in the construction industry and raise the level of potential safety and health awareness.

## 1. Introduction

Industry workers are leading national development through steady production activities. These activities have continued to grow in recent years through the incorporation of technology based on the 4th Industrial Revolution, accompanied by technological progress that exceeds the limits of human activity. However, despite the advancements achieved and the growth of technology, safety issues at industrial sites have not been resolved, thus requiring continuous research support and national policy applications. Several countries around the world have already recognized the importance of accident prevention policies.

In South Korea (henceforth, Korea), a “Five-year Occupational Accident Prevention Plan” is being implemented [1], which proposes to achieve accident prevention targets within a certain period of time. In Japan, the “Occupational Accident Prevention Plan” has been established with the aim of encouraging safety and health-related activities targeted at workers. In Germany, along with the technology policy strategy “Industrie 4.0”, “Arbeiten 4.0” is also being promoted to improve quality of work. In the UK, the Occupational Safety and Health Executive has created and implemented an annual “HSE Business Plan” to ensure the safety and health of employees [2].

Construction is an industry with a high probability of worker accidents and deaths [3,4,5,6,7]. The potential sources of these are temporary equipment and heavy equipment used during construction, as well as worker behavior and unreasonable construction project orders (insufficient construction cost or construction period) [8,9,10,11,12,13,14,15,16].

To prevent such accidents in advance, a study was conducted to reinforce the role of safety managers of suppliers with a high probability of accidents [17,18] and to explain the role of safety managers at each construction stage (client, designer, supervisor, and contractor) [19,20]. In addition, solutions incorporating state-of-the-art technologies have been proposed to mitigate against risk factors in construction projects. To prevent the risk of workers being directly exposed to hazards at a construction site, researchers have proposed technical solutions, such as applying a monitoring technique using a real-time locating system (RTLS) [21,22] or identifying safety risk factors at the design stage using BIM in multidimensional visualization [23,24,25].

However, these proposals are intended to improve construction safety during construction, as construction companies’ preemptive disaster prevention technology and construction safety policy proposals are relatively insufficient. Therefore, to overcome these limitations, it is necessary to improve state-led construction accident prevention policy so that it can work organically in consideration of the size, safety, and health capabilities of construction companies.

In Korea, the Korea Occupational Safety and Health Corporation implemented the “Evaluation of Construction Company Industrial Accident Prevention Activities” system in 2014. This system encourages voluntary accident prevention activities by construction companies and is characterized by a lack of legal compulsion and policy support. Thus, to induce voluntary participation, the system gives a maximum of +1 point for pre-qualification (PQ) and allows construction companies to receive additional points for order-taking activities for public work projects when the evaluation score is 50 points or more. Thus, a system of pre-autonomous safety and health-related activities for construction companies has been established under the guidance of state agencies.

It is obligatory to revise this system every three years according to the Occupational Safety and Health Act of Korea, and the specific revisions are dependent on the recent status of serious accidents in the construction industry and the effectiveness as evaluated by the authorities, including the Ministry of Employment and Labor of Korea and the Korea Occupational Safety and Health Agency.

**Figure 1:**
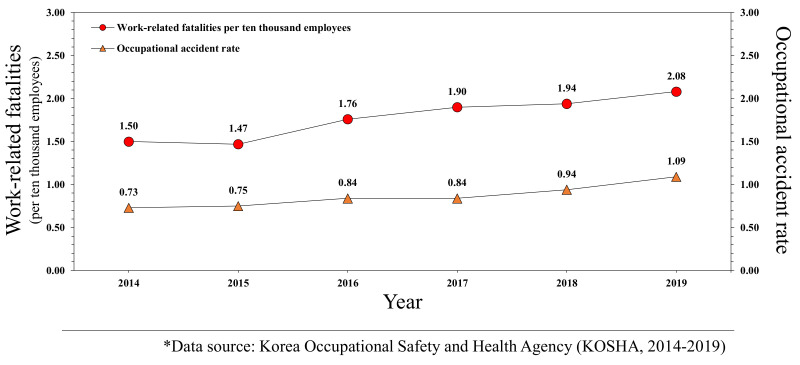
Yearly accident rate and fatality rate in the construction industry.

However, despite two revisions since 2014, there have been no reorganizations or detailed supplements in six years. In addition, there have been no significant improvements to the occupational accident rate and fatality rate in construction industries, as shown in Figure 1. Improvements to occupational safety and health are expected following the improvement of construction industry-related laws, policies, and systems as well as the introduction of advanced safety technology; nevertheless, policies and systems enhancing the safety management awareness of the companies in the construction industry should be continuously improved. One of these is the “Evaluation of Construction Company Industrial Accident Prevention Activities” system. By revising its details, the effectiveness of the system in terms of reductions in the accident and fatality rate in the construction industry can be improved. Consequently, it is required to review the effectiveness and rationality of the detailed evaluation criteria of the current system in order to suggest an effective system improvement plan which can have a direct impact on the reduction in serious industrial accidents in the construction industry.

This study aims to suggest improved evaluation details for enhancing the effectiveness of the “Evaluation of Construction Company Industrial Accident Prevention Activities” system based on an analytical approach using the evaluation data provided by the Korea Occupational Safety and Health Agency [26]. First, evaluation data for five years after the implementation of the system were analyzed, and based on the results of the analysis, a review was conducted on the system’s effectiveness and rationality. Based on this, it was possible to determine the level and status of the safety and health competency of companies participating in the evaluation and to propose a system improvement model that considers the size, safety, and health competency of the company. The validity of the proposed model was verified through a survey of construction workers (site safety manager, order-taking manager, manager, and supervisor). With the help of the aforementioned process, this study proposes measures to encourage the voluntary participation of construction companies and supervisors to increase the levels of safety and health awareness of potential companies and actively contribute to the prevention of accidents.

## 2. Review of the Current Evaluation System of Construction Companies’ Industrial Accident Prevention Activities in Korea

Safety-related policies for the construction industry have been continuously developed by the government, but despite these efforts, accident and fatality rates have not decreased significantly. Accordingly, the Korea Occupational Safety and Health Agency has been conducting an “Evaluation of Construction Company Industrial Accident Prevention Activities” since 2014 to prevent industrial accidents and protect workers from workplace hazards. The main items in this evaluation are employers’ safety and health education, inspection and participation in events, percentage of full-time employees who work as safety and health managers, safety and health organization, and Korea Occupational Safety and Health Management System (KOSHA-MS) certification, and evaluation is carried out on voluntarily participating companies.

**Figure 2:**
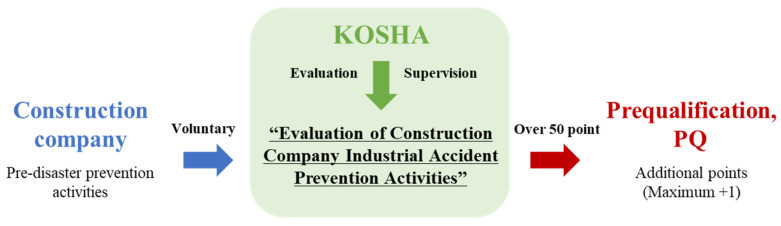
System evaluation and PQ process.

As shown in Figure 2, when a construction company bids for public works, if the evaluation score of industrial accident prevention performance is 50 points or more, this is reflected in the PQ as an additional point to promote active pre-autonomous safety activities.

### 2.1. Overview of the System

#### 2.1.1. Evaluation Target

The evaluation target of the system is a general construction company that holds a civil and building construction license and is within the first 1000 positions in the ranking of construction capacity evaluation, based on Article 23 of the “Construction Industry Framework Act” enacted by the Ministry of Land, Infrastructure, and Transport. The construction capability evaluation amount is calculated by comprehensively evaluating the construction companies’ construction performance, management status, technical capability, and reliability and is announced by the Korea Construction Association at the end of July every year. The construction companies subject to evaluation are classified into Groups 1 to 4 according to their ranking in the construction ability evaluation and are summarized in Table 1.

#### 2.1.2. Evaluation Items

According to Article 15 (safety manager, etc.) and Article 16 (health manager, etc.) of the Occupational Safety and Health Act, companies are classified as Type A (construction company with a safety and health manager appointed on duty at the construction site) and Type B (construction company without a safety and health manager appointed on duty at the construction site) and are evaluated on the basis of common items and awarded additional points. The common items are employee safety and health education, inspection and participation in events, percentage of full-time employees as safety and health managers, Safety and Health Organization certification, and an additional point for Korea Occupational Safety and Health Management System (KOSHA-MS) certification, and performance is calculated with a total of 100 points. Table 2 and Table 3 list the evaluation indexes, detailed indexes, and scores for each item. Type A is a company with a site with a building project worth more than USD 11 million or a site with a civil engineering project worth more than USD 14 million. Otherwise, it is a Type B company.

### 2.2. Status Analysis of the Current System Based on the Evaluation Results Accumulated from 2014

#### 2.2.1. Yearly Evaluation Participation Ratio and Average Point

**Figure 3:**
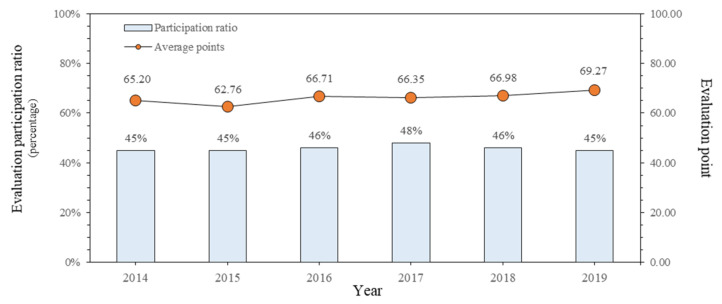
Participation ratio and average points trend by year.

The status of the annual “Evaluation of Construction Company Industrial Accident Prevention Activities” provided by the Korea Occupational Safety and Health Agency was reviewed. The number of construction companies that participated in the evaluation from 2014 to 2019 was 449 to 476 (45% to 48%) out of 1000, and the average score was calculated to be 62.76 to 69.27 out of 100 points. In Table 4, the participation rate and average score are sorted and shown in Figure 3.

The average points tend to rise as the engagement rate stays above 45% over six years. Two reasons can be predicted for this. First, it can be seen that the safety and health competency of companies participating in the evaluation may have increased, and second, the ability of the system to discriminate against evaluation items may have decreased due to the accumulated experience in evaluation. Therefore, a revision is required so that an appropriate evaluation can be carried out using this system.

**Figure 4:**
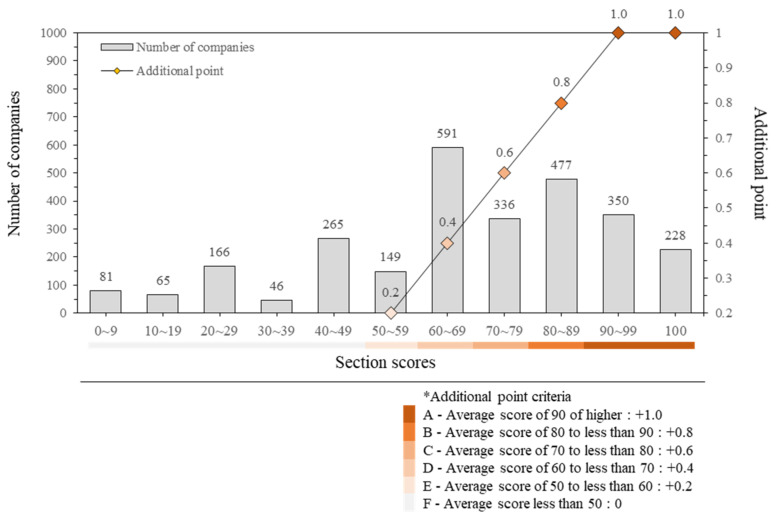
Distribution by participating companies’ evaluation points.

To determine the rate at which companies participating in this system receive additional points for PQ, evaluation points are sorted by section and shown in a graph in Figure 4. Of the 2754 companies participating in the evaluation, 2131 (77%) received additional points in PQ (50 points or more). Among the companies with an evaluation point of 50 or higher, 10.7% received the highest possible score of +1, and 7% received the lowest score of +0.2. The remaining 92.3% scored between +0.4 and +0.8.

Table 5 shows the average points for each item according to the type (Type A/Type B) of the construction companies participating in the system.

The average points for Type A companies were 70.69, and most companies earned more than 50 points, which is an additional point of PQ. In contrast, in the case of Type B companies, the average score was 49.2, indicating that most companies did not achieve additional points of PQ.

#### 2.2.2. Participation Rates and Average Points for Each Group

The system divides the companies to be evaluated into Groups 1 (1st–100th), Group 2 (101st–300th), Group 3 (301st–600th), and Group 4 (601st–1000th) according to their ranking in the construction capability evaluation. Table 6 summarizes the participation ratios and average points of each group evaluated accordingly.

**Figure 5:**
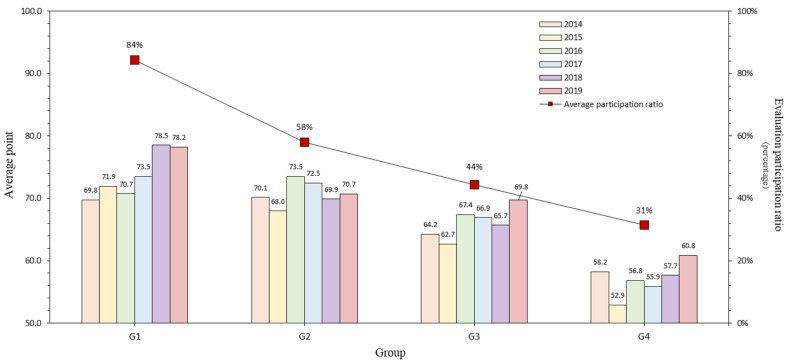
Participation ratio and average point trend by group, classified according to year.

**Figure 6:**
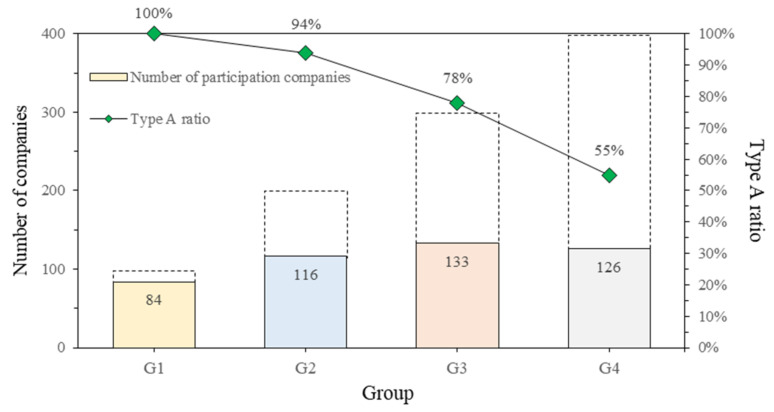
Participation and Type A holding ratio by classified group.

In Figure 5, the participation ratio and average points earned by the group are listed and plotted as a graph over six years. The graph confirms that the participation ratio and average points of the top construction companies were high. Figure 6 shows the ratio of Type A companies participating in the evaluation. Likewise, companies in the upper group showed proportional results.

Table 7 shows the average points for each item according to the type of construction companies participating in the system (Type A/Type B), classified by group.

The distribution of item average points was investigated by dividing Types A and B into groups. In the case of companies classified as Type A, which are classified as relatively large-scale workplaces, the average points for each item and the total average points are high. In addition, it was confirmed that the average number of companies in the high group was also high. In Group 1, there were no companies classified as Type B, so the average points were not counted.

### 2.3. Implications for the System Improvement

The participation ratio and average points of the companies subject to evaluation were analyzed according to year and group classification in the performance evaluation history of the system, which has been conducted by the Korea Occupational Safety and Health Agency since 2014. Consequently, the implications derived by analyzing the evaluation history of the system implemented for a total of six years can be summarized as follows:

(1) Of all general construction companies (a total of 13,000 registered companies), the number of companies that can participate is limited to 1000. Each year, more than 450 companies participate in the system, 77% of which earn extra points in PQ. Companies that do not qualify for participation cannot obtain additional points for PQ under this system; therefore, it is necessary to improve the evaluation targets to ensure equity;
(2) In the overall history of the evaluation, the average participation ratio was maintained above 45%, but the average point gradually increased. This can be predicted as an increase in the safety and health competency of the enterprises participating in the evaluation or a decrease in discrimination for the evaluation items. Therefore, it is necessary to consider the safety and health competency of the company and improve the system by increasing the number of evaluation items;
(3) The participation ratio and average point according to classification and evaluation as groups were higher in Type A, which has a higher group and a large workplace. Safety and health competency are proportional to the size of the company and the size of the workplace, and the system needs to be improved to enable level–level evaluation to take this into account;
(4) Currently, the evaluation items organized in this system encourage minimum safety awareness and safety and health activities to prevent accidents autonomously. In addition, the introduction of evaluation items for the direct prevention of industrial accidents by construction site workers is expected to have a positive effect on accident statistics related to the construction industry;
(5) Measures are needed to encourage voluntary safety and health activities of construction companies subject to evaluation and to induce active participation. To improve these aspects, efficient evaluation and the presence of a supervisory authority are also important.

## 3. Suggestions for an Improved System and Evaluation Details

The primary objective of this revision is to enhance the effectiveness of the system in terms of encouraging voluntary prevention activities of construction companies. For this, the details of the evaluation indicators and scores were revised. In addition, the classification of the evaluation target companies was significantly changed to grow the number of companies that can participate in the system.

The details of the system in effect in the Korea Occupational Safety and Health Agency and the evaluation history data were analyzed to find possibilities for improvement. Based on the derived results, an improvement model for the evaluation targets and evaluation items of the system was proposed. For effective revision, the validity of detailed evaluation indicators and evaluation target calculations was reviewed through the current status of major disasters, the participation rate of this system for the past 5 years, score statistics for each group, and the collection of stakeholder opinions via interview, survey, and public hearings.

### 3.1. Evaluation Targets and Classification Improvement

In order to encourage autonomous safety and health activities of construction companies, if a company participating in the “Evaluation of Construction Company Industrial Accident Prevention Activities” achieves a certain level of performance score or higher, it can receive additional points from PQ. In the existing system, the evaluation targets companies within the first 1000 places in the construction capability evaluation ranking of general construction companies with civil and building construction licenses; thus, 12,000 out of about 13,000 general construction companies are restricted from participating. Therefore, it is necessary to encourage all general construction companies to participate in the system, and this can be achieved if those companies obtain the qualifications necessary to participate in the evaluation. As outlined below, this is the direction of our suggested improvements, so that the increased evaluation targets can be efficiently classified, and systematic evaluation can be performed.

#### 3.1.1. Expansion of Evaluation Targets

To encourage participation in the system and to promote voluntary safety and health activities of companies, it was proposed to expand the evaluation targets from those in the first 1000 places in the construction capacity evaluation ranking of general construction companies with civil and building construction licenses to all general construction companies.

**Figure 7:**
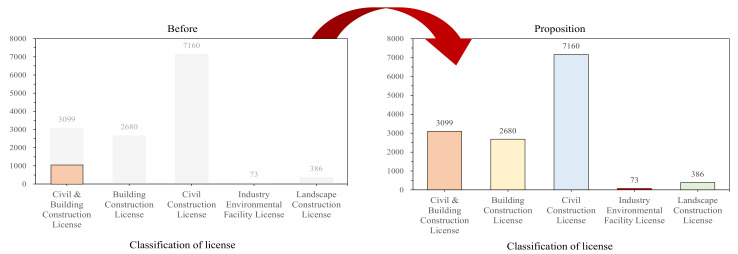
Expansion of system evaluation target qualifications (total number of construction companies: Construction Association of Korea, 2020).

Previously, the evaluation qualification criteria limited participation to those companies with civil and building construction licenses, but as shown in Figure 7, companies that would be allowed to voluntarily participate if we were to expand the evaluation target to all general construction companies can earn additional points from PQ. This is expected to alleviate the issue of equity between companies and increase the number of participants.

#### 3.1.2. Improvements to the Classification of Evaluation Targets

In the new target classification system, the classification criteria from Groups 1 (G1) to 4 (G4) classified by the construction capacity evaluation ranking were used to divide companies based on integrated construction capacity evaluation amount ranking and reorganize them into Evaluation Groups 1 (EG1) through 3 (EG3), as shown in Table 8.

Evaluation Group 1 (EG1) consists of companies with high interest in voluntary participation in safety and health activities set by the Korea Occupational Safety and Health Agency for Harm and Hazard Prevention Plan system.

Evaluation Group 2 (EG2) consists of companies that have participated in the existing system and have sufficient evaluation experience, safety, and health performance.

Evaluation Group 3 (EG3) consists of companies that plan to participate in the expansion of evaluation targets. To apply more comfortable indicators than other evaluation groups, Evaluation Group 3 was composed of new participating companies and small companies.

As the number of companies to be evaluated increases, the purpose of the proposed three evaluation groups is to prevent confusion in the evaluation and to facilitate the evaluation and management of supervisory agencies.

### 3.2. Improvement of Evaluation Items

Previously, the scope of participation in the evaluation target of the system was expanded, and a new classification system was proposed. Accordingly, detailed evaluation items were improved in consideration of the overall size, safety, and health capabilities of general construction companies, and evaluation items that positively influence the accident index of the construction industry were developed.

#### 3.2.1. Improvement of Common Items

- Employers’ safety and health education, inspection, and participation in events
  - Because the completion of safety and health education is extended to all general construction companies, it is proposed that the employer can be provided online or offline education for smooth evaluation, as shown in Table 9;
  - Participation in on-site safety and health inspection and safety inspection day events was changed to on-site safety inspection and improvement measures, as shown in Table 10. Therefore, it was proposed to prevent potential worker hazards in advance by safety inspections being conducted on-site by the employer and implementing practical improvement measures;
- Percentage of full-time employees who are safety and health managers (only Type A)
  - According to evaluation history, most companies classified as Type B among the top groups achieved an average of 35 points or more out of 40 points in the previous evaluation. In addition, even if the proportion of regular workers is less than 20%, a basic score of 15 points can be obtained, so discrimination against evaluation has decreased. Therefore, the ratio of regular workers increased, and the points allocated were adjusted, as shown in Table 11;
- Safety and health organization
  - The evaluation items and scores were organized according to the newly configured evaluation group classification system for evaluation items classified based on the construction capability evaluation amount ranking from Group 1 to Group 4. Considering the level of companies classified from Evaluation Group 1 to Evaluation Group 2, a formula for calculating the score of the safety and health organization was proposed, and for Evaluation Group 3, the minimum evaluation items for safety and health organization were applied (As shown in Table 12).
- Efforts to reduce accidents and fatalities
  The accident fatality reduction effort indicator is a newly constructed evaluation indicator to reduce serious accidents that cause worker deaths during construction through participation in the system. Therefore, to effectively reduce major accidents in the construction industry, proposals were made for each type of construction company, as shown in Table 13.

#### 3.2.2. Improvement of Additional Points

- Korea Occupational Safety and Health Management System (KOSHA-MS) certification
  - Only companies with sufficient safety and health competency and voluntary preventive efforts are eligible for KOSHA-MS certification (maintaining), and the Korea Occupational Safety and Health Agency performs the application and certification process. Therefore, it is suggested to verify that companies have sufficient safety and health capabilities and to maintain the existing items as items that provide additional points to companies that have performed appropriate certification procedures, as shown in Table 14.
- Safety and health-related award performance
  - The safety and health-related awards were not reflected in the additional points because the previous study expected fewer awards. However, in recent years, the importance of disaster-related issues, safety, and health has increased, and companies are conducting voluntary research and technology development related to safety and health. To reflect the voluntary efforts of companies as a result of awards related to safety and health, a new additional point index, as shown in Table 15, was proposed.

### 3.3. Survey about the Suggested System and Evaluation Details

To verify the validity of the new evaluation of construction companies’ industrial accident prevention activities proposed in this study, a questionnaire survey was conducted on the degree of awareness of the existing system, improvement model for the evaluation target, and improvement model for detailed evaluation items. The survey was conducted on various stakeholders, including workers in the HSE part of construction companies, owners of companies, labor unions, business associations, governmental agencies, and university professors in the construction safety engineering field. Among the construction companies registered with the Ministry of Land, Infrastructure, and Transport following Article 9 of the Framework Act on Construction Industry, all general construction companies with civil and building construction, building construction, civil construction, industry environmental facility, and landscape construction licenses were selected for the survey. The survey was conducted using a Google form, and the questionnaire was distributed via the Korea Occupational Safety and Health Agency, Construction Association of Korea, Korean Society of Safety, Korea Temporary Equipment Association, and the Korean Society of Hazard Mitigation for about 30 days.

#### 3.3.1. Questionnaire

The questions in the questionnaire were divided into main and subcategories, as shown in Table 16; the questionnaire was developed, and a sample of the questionnaire is shown in Table 17.

#### 3.3.2. Survey Results

**Figure 8:**
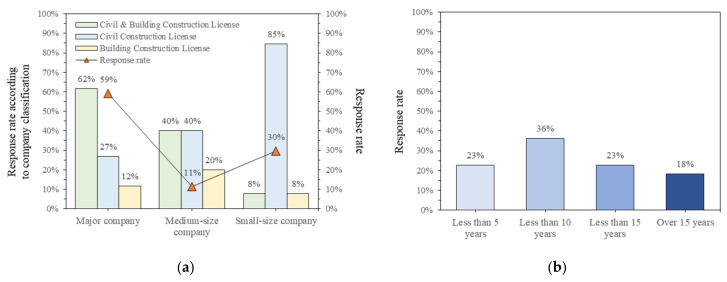
Surveyor’s basic information: (**a**) classification and license of surveyor’s company; (**b**) surveyor’s career length.

Opinions on the proposed improvement model study were analyzed using survey responses distributed through five organizations for all general construction companies. Regarding the size of the companies belonging to the survey respondents, 59% of the total respondents were large companies, 11% mid-sized companies, and 30% small and medium-sized companies, and the status of license holdings for affiliated companies is shown in Figure 8a. Figure 8b shows the industry experience of the survey.

**Figure 9:**
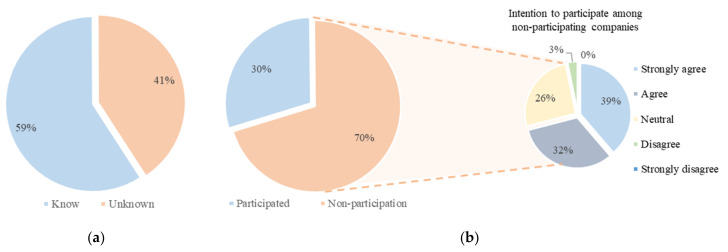
Survey results on existing systems: (**a**) recognition of the existing system; (**b**) experience and intention to participate in the existing system.

In Figure 9a, we can see that 59% answered “know” in the survey to the question regarding whether they knew the system. In addition, the survey question regarding whether they participated in the system, the percentage of nonparticipation was found to be 70%. Among nonparticipating companies, 97% of all respondents with “neutral” or higher expressed willingness to participate in the future, as shown in Figure 9b.

**Figure 10:**
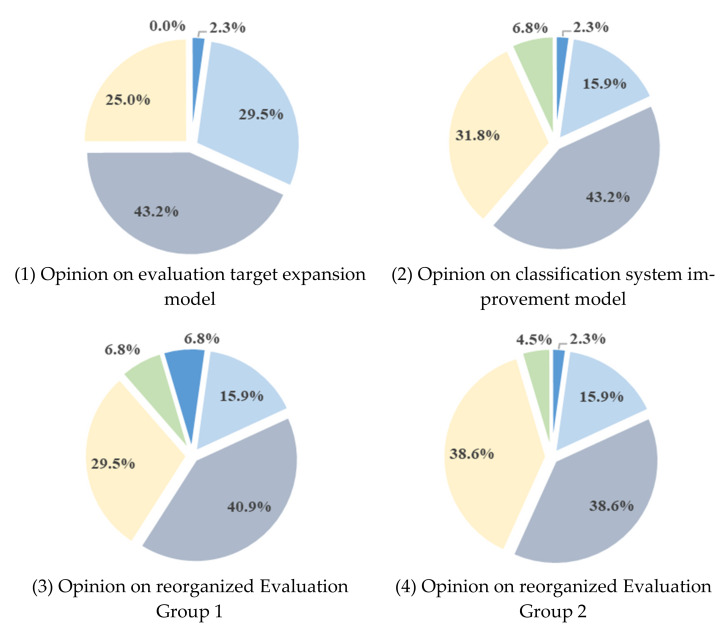

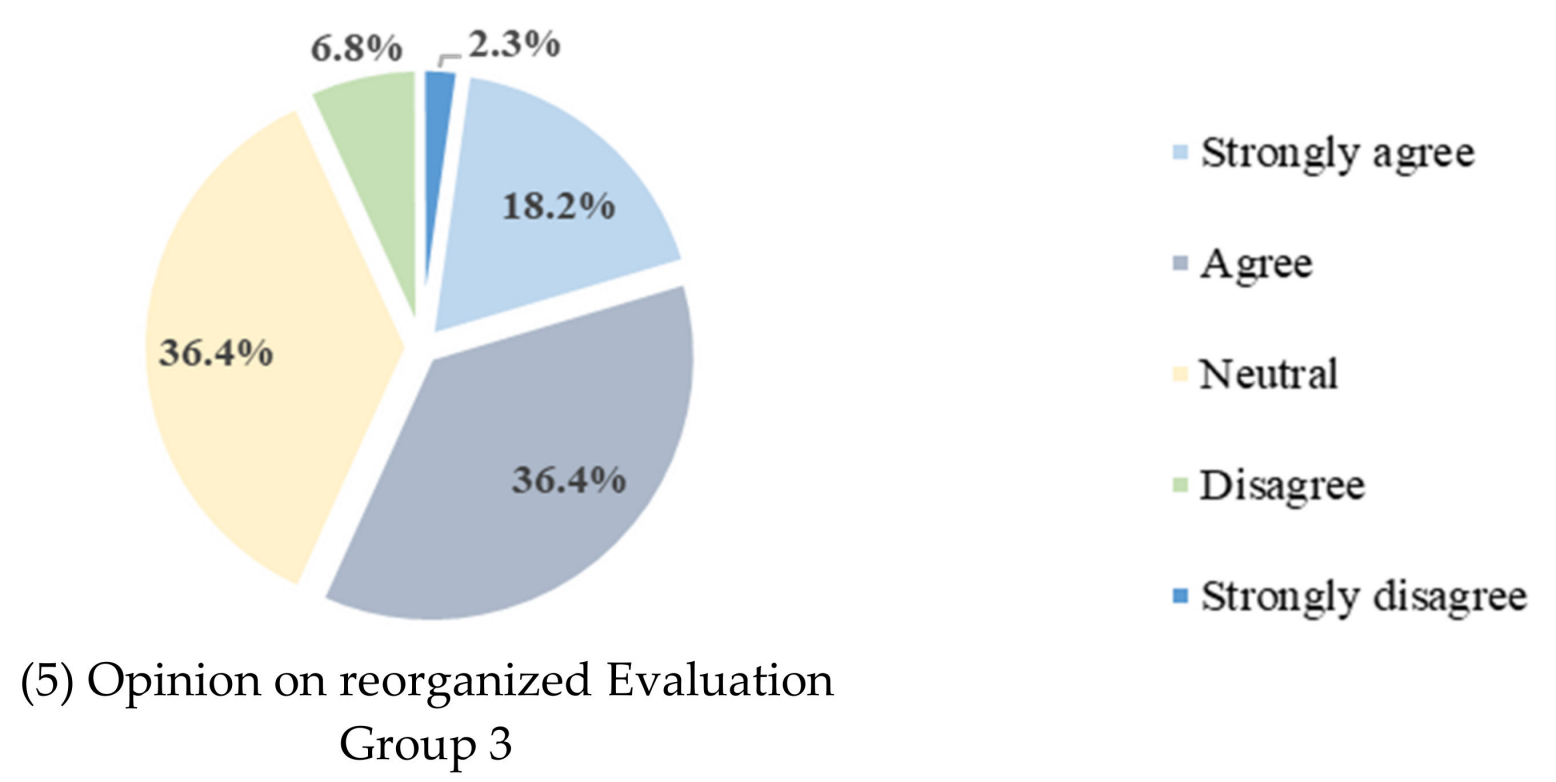
Survey results for evaluation targets and the classification improvement model.

Figure 10 shows the questionnaire survey on the model for expanding the evaluation targets and improving the classification system proposed in this study. (1) In the expanded opinion of the evaluation target, more than 72.7% answered “agree”, 25% answered “neutral”, and 2.3% answered “disagree”. (2) Regarding opinions about whether the classification system required improvement, 59.1% answered “agree”. With regard to the proposed classification system, more than 54% of the responses from Evaluation Groups 1 to 3 were positive.

**Figure 11:**
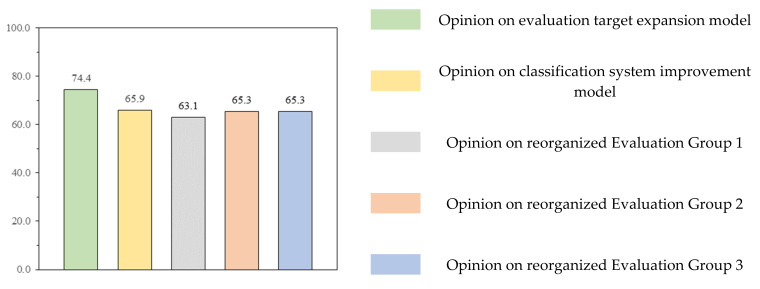
Survey weighted score for evaluation targets and the classification improvement model.

In Figure 11, the score for each questionnaire item is calculated by applying weights to opinions as expressed through survey responses. In addition, there were answers such as “If this system is expanded to all general construction companies, there will be an effect of preventing industrial accidents”, “It is possible to acquire additional PQ points that were not previously received”, and “The existing criteria for evaluation were unreasonable. It is reasonable to expand the evaluation target” as comments on the expanded evaluation target. As regards comments on the improvement of the classification system, some of the answers were “It is necessary to actively promote the improvement model”, “It should be improved to take into account the size of the company and its safety and health capabilities”, and “It is necessary to sequentially reflect the improved model”. Opinions on improvement from EG1 to EG3 showed a score of 60 or more on a weighted average scale, and most of the participants from these groups offered positive answers.

**Figure 12:**
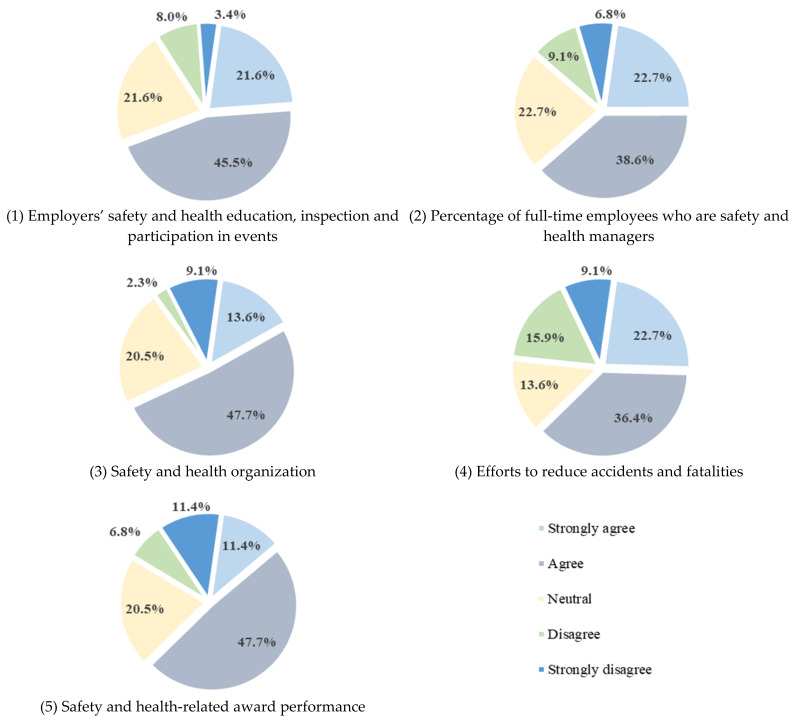
Survey results for each item improvement model.

Figure 12 shows the results of the survey on the improvement model by item. Regarding the common item, 67.1% of the respondents expressed a positive opinion regarding the improvement of employers’ safety and health education, inspection, and participation in events. The percentage of full-time employees appointed as safety and health managers is a model that reinforces the existing evaluation indicator (increasing the proportion of regular workers and removing the basic point), and 61.3% answered positively in the questionnaire. A total of 61.3% responded with positive opinions regarding the item on “safety and health organization”, which was proposed to enable evaluation at the enterprise level by applying the improved classification system. As regards opinions on the item on “efforts to reduce accidents and fatalities”, which was newly formed, 59.1% answered positively. In the questionnaire on additional points, 59.1% answered positively to the newly proposed “safety and health-related awards” performance.

**Figure 13:**
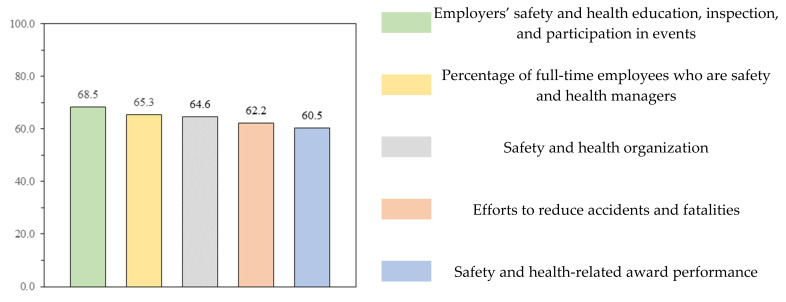
Survey weighted score for each item improvement model.

In Figure 13, the average score converted to weight was 60 or higher. Table 18 summarizes the comments and opinions of each questionnaire item for each improvement model.

## 4. Conclusions

Voluntary safety and health activities are encouraged by reflecting the evaluation results of construction companies participating in “Evaluation of Construction Company Industrial Accident Prevention Activities” in PQ. The evaluation targets of the system are classified into Groups 1 to 4 in the first 1000 places in the ranking of construction capacity evaluation of general construction companies with civil and building construction licenses. The performance evaluation is classified into Types A and B according to the presence or absence of a workplace where a safety and health manager should be appointed and is evaluated by item.

From the performance evaluation data collected for six years, the participation rate and performance evaluation points of the companies participating in the system were analyzed by being divided by year, group classification, and evaluation items. As a result of the analysis, it was found that the higher the rank of construction capacity evaluation or the higher the percentage of possession of Type B was, the higher the performance evaluation score. This indicates that the economic scale of a company and the ratio of large-scale workplace holdings represent safety, health competency, and level. Therefore, if the system taking this into account is improved and effective disaster prevention evaluation items are supplemented, usability can be maximized.

In this study, a model for improving the system was proposed, and the appropriateness of the model was analyzed through a questionnaire survey of people related to general construction companies. In addition, this suggests an effective application direction for the improvement model.

(1) Suggestions regarding evaluation target and classification adjustment:
  - The existing evaluation target was limited to 1000 general construction companies with civil and building construction licenses. In the proposed model, evaluation targets are expanded, and qualifications are granted so that all general construction companies can participate;
  - In the case of improvement of the classification system, the experience of participating in the evaluation and the safety and health competency of companies with participation qualifications were considered. This led to the proposed classification into EG1, EG2, and EG3 as the ranking of the total construction capability evaluation (total construction capacity evaluation number of licenses held by general construction companies);
  - The results of the survey of relevant parties are shown in Figure 10 and Figure 11, and the positive rate for expanding the evaluation target was 72.7% (weight score 74.4), while the positive rate for improving the classification system was 59.1% (weight score 65.9). Most of the respondents gave positive answers in the questionnaire regarding the expansion of evaluation targets and the classification system improvement model.
(2) Suggestions regarding evaluation item improvement:
  - To expand the evaluation target and satisfy the classification system improvement model, an evaluation item improvement model is proposed. Existing common items and additional points have been improved, and assigned points have been changed in consideration of the size of the company and the level of safety and health management;
  - Among the most common items, “Employers’ safety and health education, inspection, and participation in events” was used to encourage actual improvement measures after online/offline parallel education of the employer and employers’ on-site safety and health check. “Percentage of full-time employees who are safety and health managers” increased the rate of hiring regular workers and removed the basic points to increase discrimination. In “safety and health organization”, the calculation formula was developed in consideration of the ranking of the integrated construction capability evaluation amount of construction companies and safety and health capability. To directly reduce fatal accidents in the construction industry, “efforts to reduce accidents and fatalities” is classified as a license held by a construction company, and an evaluation indicator is presented;
  - As regards additional points, “safety and health-related award performance” was added, while the “Korea Occupational Safety and Health Management System (KOSHA-MS) Certification” was kept in order to encourage voluntary safety and health activities, as well as research and technology development by companies;
  - The survey results are shown in Figure 12 and Figure 13, and in the multiple-choice question on the proposed evaluation item improvement model, more than 59.1% (weight score 60.5) of the responses were positive. Table 18 summarizes the short-answer opinions of the questionnaire survey based on a 50-point weight, and the answers are focused on the evaluation and supplementation of the proposed improvement model.
(3) Suggestions regarding improving implementation:
  - In this study, the expansion and classification of evaluation targets were improved to encourage the participation of more construction companies, and an effective level evaluation and accident reduction model was proposed to improve the evaluation items;
  - The system is operated as a system that encourages construction companies to voluntarily participate in disaster prevention activities, so there is no role or economic support as a legal device designated and implemented by the state. Thus, if the performance evaluation score is 50 or more, a maximum of +1 points is given to the preliminary screening of the bid participation qualification, reflecting the efforts of participating companies to prevent accidents. Therefore, in order for construction companies to voluntarily participate in the system improvement model, additional points for PQ should be offered, and the performance evaluation scores should be reflected in the national public work bidding system;
  - The supervisory authority must provide evaluation solutions and manuals for each company to be evaluated, promote active public relations activities, hire evaluation personnel, and establish an evaluation system for accurate evaluation;
  - In future, if the evaluation data of this system are analyzed and the evaluation items are continuously improved along with R&D, it will contribute to active accident prevention and improvement of safety and health awareness of construction companies.

It is obligatory for this system to be revised every three years based on the recent status of serious accidents in the construction industry and the effectiveness as evaluated by the authorities. This means that if the suggested model is adopted and imposed, the effectiveness of this model should be evaluated 3 years later. Therefore, it is expected that the effectiveness of the suggested model could be investigated based on the change of the status and trends of occupational accident and fatality rates, participation rates, and the detailed scoring results of the participating companies.

## Figures and Tables

**Table 1:** Classification criteria for evaluation targets.

Classification	Construction Capacity Evaluation Amount Ranking
G1	1st–100th
G2	101st–300th
G3	301st–600th
G4	601st–1000th

**Table 2:** Construction companies with construction sites where are obligated to appoint a safety and health manager (Type A).

Item	Evaluation Indicators	Detailed Indicators	Points
Common item (100 points)	1. Employers’ safety and health education, inspection, and participation in events (40 points)	Completion of safety and health education	25
		Participation in on-site safety and health inspection, safety inspection day event	15
	2. Percentage of full-time employees who are safety and health managers (40 points)	Percentage of full-time employees who are safety and health managers	
		- Over 60%	40
		- Over 50%	35
		- Over 40%	30
		- Over 30%	25
		- Over 20%	20
		- Less than 20%	15
	3. Safety and health organization (20 points)	Varies according to the organization’s standards for safety and health organization.	0–20
Additional points (5 points)	Korea Occupational Safety and Health Management System (KOSHA-MS) certification (5 points)	In case of obtaining or maintaining certification	5
		Applying for certification	2
Total	105 points (Maximum 100 points)		

**Table 3:** Construction companies without construction sites where are obligated to appoint a safety and health manager (Type B).

Item	Evaluation Indicators	Detailed Indicators	Points
Common item (100 points)	a. Employers’ safety and health education, inspection, and participation in events (50 points)	Completion of safety and health education	25
		Participation in on-site safety and health inspection, safety inspection day event	25
	b. Safety and health organization (50 points)	Varies according to the organization’s standards for safety and health organization	0–50
Additional points (5 points)	Korea Occupational Safety and Health Management System (KOSHA-MS) certification (5 points)	In case of obtaining or maintaining certification	5
		Applying for certification	2
Total	105 points (Maximum 100 points)		

**Table 4:** Average points of evaluation performance by year.

Year	2014	2015	2016	2017	2018	2019
Total participation in evaluation (EA)	449 /1000	453 /1000	464 /1000	476 /1000	461 /1000	451 /1000
Participation ratio (%)	44.9	45.3	46.4	47.6	46.1	45.1
Average points (Point)	65.20	62.76	66.71	66.35	66.98	69.27
Standard deviation (Point)	24.02	25.59	24.30	24.83	25.97	25.20

**Table 5:** Average points for each evaluation item according to classification.

Evaluation Type	Common Item			Additional Points	Total
	Employers’ Safety and Health Education, Inspection, and Participation in Events	Percentage of Full-Time Employees Who Are Safety and Health Managers	Safety and Health Organization	Korea Occupational Safety and Health Management System (KOSHA-MS) Certification	
Type A	28.06 /40	35.39 /40	6.96 /20	0.28 /5	70.69 /100
Type B	30.07 /50	-	19.13 /50	-	49.2 /100

**Table 6:** Average points of evaluation performance by group classified according to year.

Year	2014				2015				2016				2017				2018				2019				Total Average			
	G1	G2	G3	G4	G1	G2	G3	G4	G1	G2	G3	G4	G1	G2	G3	G4	G1	G2	G3	G4	G1	G2	G3	G4	G1	G2	G3	G4
Total participation in evaluation (EA)	82 /100	114 /200	137 /300	116 /400	82 /100	111 /200	126 /300	134 /400	83 /100	116 /200	142 /300	123 /400	86 /100	119 /200	136 /300	135 /400	84 /100	123 /200	130 /300	124 /400	89 /100	113 /200	128 /300	121 /400	84 /100	116 /200	133 /300	126 /400
Participation ratio (%)	82	57	46	29	82	56	42	34	83	58	47	31	86	60	45	34	84	62	43	31	89	57	43	30	84	58	44	32
Type A ratio (%)	100	92	70	45	100	94	71	51	100	95	77	52	100	94	82	58	100	93	81	60	100	97	84	63	100	94	78	55
Average points (Point)	70	70	64	58	72	68	63	53	71	74	67	57	73	72	67	56	79	70	66	58	78	71	70	61	74	71	66	57
Standard deviation (Point)	19	23	25	25	20	20	27	28	20	20	25	26	18	22	26	27	18	22	28	29	17	23	25	30	19	22	26	28

**Table 7:** Average score for each evaluation item by group.

Classification and Evaluation Type		Common Item			Additional Points	Total
		Employers’ Safety and Health Education, Inspection and Participation in Events	Percentage of Full-Time Employees Who Are Safety and Health Managers	Safety and Health Organization	Korea Occupational Safety and Health Management System (KOSHA-MS) Certification	
Group 1	Type A	31.67 /40	31.70 /40	9.33 /20	1.19 /5	73.88
	Type B	-	-	-	-	-
Group 2	Type A	28.49 /40	36.85 /40	7.03 /20	0.01 /5	72.38
	Type B	28.87 /50	-	17.84 /50	0 /5	46.71
Group 3	Type A	27.36 /40	36.76 /40	6.80 /20	0 /5	70.92
	Type B	29.24 /50	-	20.89 /50	0 /5	50.14
Group 4	Type A	24.05 /40	35.56 /40	4.25 /20	0 /5	63.86
	Type B	30.78 /50	-	18.40 /50	0 /5	49.17

**Table 8:** Proposal of classification system for evaluation targets.

**Before**		**➪**	**Proposition**	
**Classification**	**Construction Capacity Evaluation Amount Ranking**		**Classification**	* Integrated Construction Capacity Evaluation Amount Ranking
G1	1st–100th		EG1	1st–300th
G2	101st–300th		EG2	301st–1000th
G3	301st–600th		EG3	All general construction companies below 1001st
G4	601st–1000th			

* Integrated construction capability evaluation amount: sum of construction capacity evaluation number for all licenses held by general construction companies.

**Table 9:** Evaluation details for completion of safety and health education.

**Before**			**➪**	**Proposition**		
**Detailed Indicators**	**Evaluation Type**	**Points**		**Detailed Indicators**	**Evaluation Type**	**Points**
Completion of safety and health education	Type A	25		Completion of safety and health education	Type A	15
	Type B	25			Type B	25
Completion of offline education for employer				Completion of online and offline education for employers		

**Table 10:** Evaluation details for on-site safety inspection and improvement measures.

**Before**			**➪**	**Proposition**		
**Detailed Indicators**	**Evaluation Type**	**Points**		**Detailed Indicators**	**Evaluation Type**	**Points**
Participation in on-site safety and health inspection, safety inspection day event	Type A	15		on-site safety inspection and improvement measures	Type A	25
	Type B	25			Type B	25
Employers’ inspection and event participation Once a month (2.5 points), Total 10 times (10 months)				Employers’ inspection and improvement measures Once a month (2.5 points). In cases of additional inspection, 0.5 points are given only once a month. Up to 3 points/month given when employers perform safety and health checks and improvement measures at two or more different construction sites		

**Table 11:** Evaluation details for percentage of full-time employees who are safety and health managers.

**Before**		**➪**	**Proposition**	
**Detailed Indicators**	**Points**		**Detailed Indicators**	**Points**
Percentage of full-time employees who are safety and health managers over 60%	40		Percentage of full-time employees who are safety and health managers over 70%	25
Percentage of full-time employees who are safety and health managers over 50%	35		Percentage of full-time employees who are safety and health managers over 60%	20
Percentage of full-time employees who are safety and health managers over 40%	30		Percentage of full-time employees who are safety and health managers over 50%	15
Percentage of full-time employees who are safety and health managers over 30%	25		Percentage of full-time employees who are safety and health managers over 40%	10
Percentage of full-time employees who are safety and health managers over 20%	20		Percentage of full-time employees who are safety and health managers over 30%	5
Percentage of full-time employees who are safety and health managers less than 20%	15		Percentage of full-time employees who are safety and health managers less than 30%	0

**Table 12:** Evaluation details for safety and health organization.

**Before**				**➪**	**Proposition**			
**Classification**	**Evaluation Indicators**	**Evaluation Type**	**Points**		**Classification**	**Evaluation Indicators**	**Evaluation Type**	**Points**
G1 (1st–100th)	1. In cases where the head office has a dedicated safety and health organization and there are 5 or more employees in charge of safety and health work, including an executive who is dedicated only to safety and health work.	Type A	20		EG1 (1st–300th)	Formula for calculating point for the safety and health organization $=\frac{m}{5-\frac{2}{299}\times \left(n-1\right)}\times 10$	Type A	0–10
		Type B	50					
	2. In cases where the head office has a dedicated safety and health organization and there are 5 or more employees in charge of safety and health work.	Type A	10			Formula for calculating point for the safety and health organization $=\frac{m}{5-\frac{2}{299}\times \left(n-1\right)}\times 20$	Type B	0–20
		Type B	25					
G2 (101st–300th)	1. In cases where the head office has a dedicated safety and health organization and there are 3 or more employees in charge of safety and health work.	Type A	20		EG2 (301st–1000th)	Formula for calculating point for the safety and health organization $=\frac{m}{3-\frac{2}{699}\times \left(n-301\right)}\times 10$	Type A	0–10
		Type B	50					
	2. In cases where the head office has a 2 or more employees in charge of safety and health work, including 1 qualified as a safety and health manager.	Type A	10			Formula for calculating point for the safety and health organization $=\frac{m}{3-\frac{2}{699}\times \left(n-301\right)}\times 20$	Type B	0–20
		Type B	25					
G3 (301st–600th)	1. In cases where the head office has a 2 or more employees in charge of safety and health work, including 1 qualified as a safety and health manager.	Type A	20		EG3 (below 1001st)	1. In the case where the head office has a 1 or more employees in charge of safety and health work, including 1 qualified as a safety and health manager.	Type A	10
		Type B	50					
	2. In cases where the head office has a 1 or more employees in charge of safety and health work, including 1 qualified as a safety and health manager.	Type A	10				Type B	20
		Type B	25					
G4 (601st–1000th)	1. In cases where the head office has a 1 or more employees in charge of safety and health work, including 1 qualified as a safety and health manager.	Type A	20			2. In the case where the head office has a 1 or more employees in charge of safety and health work.	Type A	5
		Type B	50					
	2. In cases where the head office has a 1 or more employees in charge of safety and health work.	Type A	10				Type B	10
		Type B	25					

m: number of employees in charge of safety and health work, including one qualified safety and health manager at the head office. n: integrated construction capability evaluation ranking. The formulas for calculating points for the safety and health organization cannot exceed the maximum points.

**Table 13:** Evaluation details for efforts to reduce accidents and fatalities.

Classification		Evaluation Indicators			Evaluation Type	Points
Civil and Building Construction License/ Building Construction License	System scaffold use performance	System scaffold use performance			Type A	0 – 25
		=	Number of private construction sites with system scaffolding installed	×35		
			Number of private construction sites			
		System scaffold use performance			Type B	0 – 30
		=	Number of private construction sites with system scaffolding installed	×45		
			Number of private construction sites			
Civil Construction License/Industry Environmental Facility License/Landscape Construction License	① Construction machinery and equipment inspection performance	Construction machinery and equipment inspection performance			Type A	0 – 25
		=	Number of construction sites that inspected 5 major construction equipment	×35		
			Number of construction sites			
		Construction machinery and equipment inspection performance			Type B	0 – 30
		=	Number of construction sites that inspected 5 major construction equipment	×45		
			Number of construction sites			
	② Fall disaster prevention efforts	Fall disaster prevention efforts			Type A	0 – 25
		=	Number of construction sites using aerial work platforms or ladder-type work platforms	×35		
			Number of construction sites			
		Fall disaster prevention efforts			Type B	0 – 30
		=	Number of construction sites using aerial work platforms or ladder-type work platforms	×45		
			Number of construction sites			

Five major types of construction equipment—excavators, truck, mobile crane, vehicle-mounted aerial platform, forklift. The formulas for calculating points for safety and health organization cannot exceed the maximum points.

**Table 14:** Evaluation details for KOSHA-MS certification.

Evaluation Indicators	Points
○ In case of obtaining or maintaining certification	5
○ Applying for certification	2

**Table 15:** Evaluation details for safety and health-related award performance.

Evaluation Indicators	Points
○ Safety and health-related award performance Award achievement 1 point, maximum 3 points	0–3

**Table 16:** Survey items by classification.

Main Category	Subcategory	Development of Survey Item
Common	(1) Company information	Classification of company size
		Classification of license
	(2) Surveyor information	Personal history
	(3) Questions related to the existing system	Recognition of existing system
		Existing system participation experience
Evaluation targets and classification improvement model	(1) Expanding evaluation targets	Opinion on the evaluation target expansion model
	(2) Classification for evaluation targets	Opinion on the classification system improvement model
		Opinion on the reorganized Evaluation Group
Improvement model of evaluation items	(1) Common item improvement model	Employers’ safety and health education, inspection and participation in events
		Percentage of full-time employees who are safety and health managers
		Safety and health organization
		Efforts to reduce accidents and fatalities
	(2) Additional points improvement model	Safety and health-related award performance

**Table 17:** Example of a survey on the improvement model for evaluation target.

Survey—Evaluation of the construction company’s industrial accident prevention activities questionnaire on the improvement model subject to evaluation										
□ Evaluation targets and classification improvement model										
- Expanding evaluation targets										
Before							➪	Proposition		
Within the first 1000 places in the construction capacity evaluation ranking of general construction companies with a civil and building construction license								All general construction companies		
- Classification for evaluation targets										
Before							➪	Proposition		
Classification	Construction capacity evaluation amount ranking							Classification		* Integrated construction capacity evaluation amount ranking
G1	1st–100th							EG1		1st–300th
G2	101st–300th							EG2		301st–1000th
G3	301st–600th							EG3		All general construction companies below 1001st
G4	601st–1000th									
* Integrated construction capability evaluation amount: sum of construction capacity evaluation number for all licenses held by general construction companies										
□ Survey content										
- What is the opinion of the questionnaire on the proposal to expand the evaluation target of Evaluation of Construction Company Industrial Accident Prevention Activities to all general construction companies?										
Answer	①	Strongly Agree	②	Agree	③	Neutral	④	Disagree	⑤	Strongly disagree
Weight	100		75		50		25		0	
Comments with a score of 50 or higher										
Comments with less than 50 score										
- Evaluation Group 1 (EG1) consisted of companies with high interest in voluntary participation in safety and health activities as the targets of Korea Occupational Safety and Health Agency to Harm and Hazard Prevention Plan system. What is your opinion on this?										
Answer	①	Strongly Agree	②	Agree	③	Neutral	④	Disagree	⑤	Strongly disagree
Weight	100		75		50		25		0	
Comments with a score of 50 or higher										
Comments with less than 50 score										

**Table 18:** Additional comments on each item of the improvement model.

Survey Content	Comments with a Score of 50 or Higher	Comments with Less than 50 Score
(1) Employers’ safety and health education, inspection, and participation in events	∙ Measures must be prepared to enable accurate evaluation of the company to be evaluated	∙ Due to misuse of proxy attendance, online education is insufficient
	∙ Online/offline education is effective if appropriate contents are developed	∙ The number of offline education lefts should be higher
(2) Percentage of full-time employees who are safety and health managers	∙ Since the adjustment in the proportion of regular workers is a big change, it needs to be implemented gradually	∙ In reality, it can be difficult for all companies to offer full-time jobs
	∙ It is reasonable to adjust the proportion of regular workers and distribute them	∙ It can be difficult to secure a full-time ratio of top construction companies due to the large number of health and safety managers employed
(3) Safety and health organization	∙ There is a need for evaluation, reflecting the size of the company and legal regulation on the organizational structure of safety and health	∙ In the case of newly participating companies, obtaining a certificate from the organization of safety and health can be difficult
(4) Efforts to reduce accidents and fatalities	∙ This will reduce fatal accidents and improve disaster indicators	∙ It can be difficult to count and evaluate performance
	∙ It is also important to ensure the safety of the system scaffolding and to supervise it during installation	∙ It is necessary to recruit personnel from supervisory authorities
	∙ An indicator that considers the features of all construction sites, legal regulations, and the role of supervisors is important	∙ Fair evaluation must be accurate and reliable
		∙ A policy to support costs related to the indicator is needed
(5) Safety and health-related award performance	∙ It is difficult to award this, but it is reasonable as an additional point indicator	∙ It is necessary to secure objectivity and reliability for award performance
	∙ It is effective in expanding recognition of the importance of awards related to safety and health	∙ For companies with many construction sites, the frequency of accidents is high, making it difficult to win awards
		∙ A thorough verification by the supervisory authority is required for determining performance worthy of an award

## Data Availability

Data are contained within the article.
